# Position biases in sequential location selection: Effects of region, choice history, and visibility of previous selections

**DOI:** 10.1371/journal.pone.0276207

**Published:** 2022-10-14

**Authors:** Ronald Hübner

**Affiliations:** University of Konstanz, Konstanz, Germany; University of Catania, ITALY

## Abstract

In some situations, e.g., when filling out lottery tickets, it can be advantageous to select random locations. However, people usually have difficulties with this, because they are biased by preferences for certain regions, such as the center of an area. According to ideas from art theory, the preferred regions reflect the hidden structure of perceptual forces within an area. In the present study, these structures were investigated and modeled under different conditions for areas with square and rectangular shape. The general task was to sequentially place a number of dots at random locations in an area by clicking with the computer mouse at corresponding positions on the screen. Whereas in a single-dot condition each dot had to be placed in an empty area, the previously placed dots remained visible in a multiple-dots condition. In three experiments it was found that dots were preferentially placed at the center, the diagonals, and the principal axes. This preference was more pronounced in the single than in the multiple-dot condition. Moreover, sequential analyses revealed that dot placing was not only planned in advance, but that the participants also agreed to some extent in their sequential selections, which produced surprisingly similar sequential spatial patterns across participants, at least for the first dots. Altogether, the results indicate that people have great difficulties with the random selection of locations. Their selections are strongly affected by the attraction of specific regions, by previous selections, and by sequential habits.

## Introduction

In everyday life, we sometimes encounter situations, where we must select one of several locations. If the locations differ in relevant aspects, then the selection might be easy and based on rational grounds. But what if the locations are subjectively or even objectively equivalent? Assume, for instance, we want to grab one of the equivalent packs of the same product on a shelf in a supermarket or mark the cells on a lottery sheet. How should we proceed? A suitable strategy for choices amongst objectively equivalent options is to choose randomly [1, 2]. However, this is not what people usually do. Rather, they often choose specific positions with a higher probability than others. In menu choice, for instance, items at the beginning or end of a menu are favored [3], although mainly for vertical arrangements [4]. Bar-Hillel [5] explained this edge preference by assuming that, even though menu items are normally presented simultaneously, they are mentally processed sequentially, and that this leads to the well-known serial position effect [6].

Whereas position biases in menu choice are relatively harmless, they can have severe negative consequences in other areas. In eyewitness identification, for instance, choosing a suspect from a simultaneous live lineup, or from a two-dimensional photo array largely based on his or her spatial position can lead to misidentification with all its negative consequences [7]. Thus, for preventing such faulty decisions, knowledge about position biases is very important.

Interestingly, different from menu selection, no edge preference occurs in eyewitness identification. Rather, besides a bias to choose from the top row of photo arrays [7], edge *aversion* is observed [7, 8]. The latter bias also occurs in multiple-choice questions, where not only test makers but also test takers have the strong tendency to prefer central locations [9]. How can these contrasting results be explained? Bar-Hillel [5] noticed that the latter two domains involve competitive social interactions with others that resemble those in hide-and-seek games and assumed that this leads to strategic decisions. The decision maker tries to guess the location chosen by the hider and chooses one of the better hiding places, i.e., the central positions.

However, preference of central positions is not only observed for choices in competitive social interactions, but also in more simple situations such as grabbing a box from the shelf [10] or selecting a seat [11]. Christenfeld [10] supposed that the central-position bias in choice behavior reflects a simple rule applied automatically to minimize mental effort. Later, Bar-Hillel, Peer [12] argued that not only mental effort is minimized in perceptual-motor tasks, but also physical effort. They assumed that the central-position bias is due to reachability, i.e., central positions are chosen because they are easiest to reach and require less motor fine tuning.

These considerations show that the origin and details of the central-position bias in simple tasks without competitive social interaction remain largely unknown. A promising approach could therefore be to investigate situations that are as simple as possible. All tasks considered so far require choosing one of several items in an array. As a consequence, it is possible that additional biases from the features of the items or array contaminate pure position biases [13]. If the items are numbers, for instance, as in lottery or roulette choices, those considered as most representative [14] are often preferred [15]. Therefore, a more direct access to position biases might be possible by examining situations where a location in an empty area has to be chosen. That even an empty area has some hidden spatial structure has already been proposed by Arnheim [16, 17]. He assumed that such a structure results from attentional and/or perceptual processes, and cause that people prefer certain regions. A simple method to make the hidden structure of an area visible is to ask persons to place a dot at a random location within this area. The pattern of superimposed dots from many persons then reveals the hidden structure. This method, however, involves the perception and production of randomness, which are research topics in its own right.

### The perception and production of randomness

The question of how persons perceive and produce randomness is of general interest in areas such as psychology, economy, and art theory. To investigate this issue, different experimental subjective-randomness tasks have been developed. For instance, persons had to judge the randomness of patterns [e.g., 18], to select random positions within an array [e.g., 19], or to generate a random pattern [e.g., 20]. Moreover, different layouts have been used. Some researchers investigated the perception or generation of random sequences, mostly with symbols representing some kind of events [e.g., 21]. Others specified one- or two-dimensional areas and asked participants to randomly place dots freely within the empty area [e.g., 22], or to randomly mark cells in a grid [e.g., 19].

In the present study the production of randomness is mainly used as method for investigating position biases in two-dimensional arrays. For this objective, participants were asked to randomly place dots in a rectangular or square area. The rationale behind this method is based on results showing that persons have great difficulties with the perception and production of randomness. Therefore, their location choices should largely be affected by the hidden structure of the given area. It can be expected that people preferentially place dots in locations that are attractive in some sense [23]. Accordingly, the choice behavior should reveal the distribution of regional attraction in a given area. In addition, for uncovering such distributions, it was also of interest in the present study to investigate processes of sequential location selection. However, before reporting more details on the present aim, a short overview of related and relevant topics and results will be given.

### Sequences of events

Sequences of events occur in many areas such as gambling, stock market, or sports. For predicting future events, it is essential to know whether the underlying generation process is purely random or involves some structured mechanism. Accordingly, an important question is to what extent people can discriminate random from non-random sequences [24]. The evidence from corresponding studies suggests that the ability to discriminate is rather low. Binary sequences, for instance, are judged as random, when they alternate frequently, but not so often that they show a pattern [25, 26]. The tendency to expect frequent alternation is known as *negative recency effect* [27] and related to the gamblers fallacy [e.g., 28, 29]. Yet, a random process can produce long sequences of identical events, which, however, are usually not perceived as random. Rather, our intuition is that the properties of a random process, i.e., equiprobability and irregularity, should also be present in short sequences. This intuition leads to what Kahneman and Tversky [14] have called the *representativeness heuristic*. An observed sequence of events is considered random if it is similar to the mental prototype of randomness.

Falk and Konold [21] proposed an alternative account. In their *implicit encoding* theory, they assume that people judge the degree of randomness of a sequence based on the ease of which it can be encoded. The more difficult a sequence can be encoded or remembered, the more random it seems to be. In a similarly manner, Hahn and Warren [30] argue that biases and misperceptions of randomness may actually reflect limits of the human short-term memory and/or attention span. Additionally, however, they assume that the biases also reflect genuine aspects of our statistical environment [see also 31]. Recently, Gronchi and Sloman [32] provided evidence for the notion that both heuristics are used. People use the representativeness heuristic for evaluating the randomness of a sequence, but the encoding strategy for evaluating its regularity.

Similar biases as in the perception of randomness have also been observed for the production of randomness. In corresponding tasks participants must imagine a random process such as throwing a coin or rolling a dice and generate an appropriate symbolic sequence [e.g., 21, 26, 27, 31]. It turns out that participants tend to over-alternate between the possible outcomes, which is compatible with the representativeness heuristic. However, rather than attributing this bias to an intuitive misunderstanding of randomness, Wiegersma [33] assumed that the over-alternation results from controlling the automatic tendency for repeating the last response. Meanwhile there are several theories for explaining the perception and production of randomness in relation to binary sequences [for an overview see 24].

Random sequences can also affect the position bias. In full-length multiple-choice tests, for instance, examinees seem to assume that the positions of correct answers are balanced, and, therefore try to produce a balanced position sequence on their own [9]. Thus, repeated location selections can not only be affected by edge aversion, but also by the tendency of producing a representative sequence.

### Marking cells in a grid

A popular method for investigating the perception and production of *spatial* randomness is to have people mark cells in one- or two-dimensional grids, which is related to producing binary sequences. Ross and Weiner [34], for instance, asked their participants to produce a random pattern by marking either 16 or 64 cells in a 20×20 grid. A comparison with the prediction of a homogeneous Poisson process revealed a significant deviation for the 64-cells condition. Specifically, the produced patterns were too regular. Interestingly, such patterns are liked more than real random ones, which often look more clustered [e.g., 35].

For the 16-cells condition, Ross and Weiner [34] observed no significant deviation from a Poisson process. However, this statistical result might be due to the small number of participants in this condition. As mentioned, specific locations are usually preferred when only few locations must be marked or selected. In Roulette [5] and Lottery Games [36], for instance, the central region is preferred, while outer regions are avoided. Even though there is also some effect of representative or personally meaningful numbers such as their birthdate, age, or postal code [37], the common overall preference becomes visible if the patterns of many persons are superimposed.

Similar results have been observed in hide-and-seek games [19, 38]. In the study of Falk, Falk [19], for instance, participants had to mark one or three cells in a 5×5 grid. They chose the locations either from the perspective of competition or from that of cooperation. Furthermore, there were two control groups. One had to choose locations randomly, while the other was instructed to produce a pleasant-looking pattern. Interestingly, superimposing the marked locations of all participants in a condition did not only reveal that the distributions were inhomogeneous, but also that they were similar across conditions. If the columns and rows are labeled from A to E and 1 to 5, respectively, then the cells C3 (center) and B4 (one step left and up from the center) were mostly preferred.

### Free placement of dots

As mentioned, Arnheim [16, 17] proposed that even empty spatial areas (frames) have some hidden structure. In rectangular areas, for instance, the axes, diagonals, and the center are regions of high attraction. Palmer and his coworker provided empirical support for this conjecture [39, 40]. They presented a circle at various positions in an empty area and asked their participants to rate `how well the circle fits within the area at that position’. As a result, the mean ratings were highest at the center and fall off monotonically with the distance from it. Moreover, mean ratings were also increased along the principal axes, and the diagonals. A more indirect approach for revealing hidden forces in a spatial area is to ask people to place a single dot or multiple dots at random location within that area.

#### Single dots

One of the first studies, in which a single dot had to be placed inside an outline area, was Psotka [41]. He used several shapes, including square and rectangle, which were drawn on a sheet of paper. The participants were instructed to place a dot inside each shape either ‘at random’ or ‘in the first place that comes to mind’. Superimposing the individual dots revealed that they were preferentially placed on or near to the diagonals of the shape. A similar result was later found by Lisanby and Lockhead [22], who gave their participants a sheet of paper with a square or rectangle drawn on it and asked them to place a single dot within the frame. They observed that the locations near the diagonals and the center were preferred. Furthermore, more dots were placed in the upper half of the area than in the lower half. Accordingly, the distributions of superimposed dots were different from that produced by a homogeneous random process. Interestingly, Lisanby and Lockhead [22] found similar results when they instructed their participants to place a dot at an aesthetically pleasing location. From this coincidence they concluded that art, aesthetics, and subjective randomness are strongly related. Furthermore, they proposed a two-step process for the selection of random locations: Persons first select an aesthetic location, which is then adjusted to appear unpredictable.

#### Multiple dots

In some studies, participants had to place multiple dots within an area. Gemelli and Alberoni [42], for instance, asked their participants in their first experiment to draw 20 dots on a rectangular sheet of paper by simulating randomly falling drops of water. They observed that at least some participants placed the dots irregularly all over the paper without forming subgroups, similarly to what has been observed for marking cells [e.g., 34]. However, in a later study by Dudley [43], where participants had to place ten dots randomly in a square area, the pattern of superimposed dots shows less irregularity. Rather, the center was preferred while the outer regions were avoided.

Thus, it seems that the spatial distribution of dots depends on how many dots must be positioned within an area. For one or few dots, people prefer the center and the diagonals, whereas many dots are distributed more evenly across the area. That many dots are more evenly distributed might be due to the fact that the visible dots make an inhomogeneous distribution more evident, and people try to avoid global inhomogeneities. Alternatively, one can assume that visible dots have a repelling effect, which prevents clustering [17]. Such an account is similar to sequential inhibition [44], i.e., the hypothesis that an already placed dot inhibits the placement of another dot in its near neighborhood, an effect analogous to the *negative recency* effect in producing random sequences [28].

### The present study

The general aim of the present study was to use subjective randomness for investigating position biases in two-dimensional areas. For this objective three experiments were conducted in which participants were asked to place dots at random locations within a given area. Based on previous results, it was expected that people are unable to do so, because a hidden structure of perceptual forces in an area makes regions differently attractive for selection. Up to now, the distribution of regional attraction was mostly investigated in experiments in which a single or multiple dots had to be placed with a pencil on a blank sheet of paper [e.g., 22, 41–43].

The present study went further and not only collected data with computers to minimize possible effects of reachability, but also examined sequential effects and the preference order of regions. For this objective, participants had to randomly place a dot in an empty area on the screen not just once, but several times in a row. For comparison, there was also a multiple-dots condition. More specifically, the first and last experiment included two basic experimental conditions. In a *single-dot condition*, each of several dots had always to be placed in an empty area, whereas in a *multiple-dots condition* the already placed dots remained visible. Furthermore, the number of dots and the form of the area were varied across experiments.

As we have seen, if participants have to place a single dot in an empty area, they prefer to choose a place near the center or diagonals. Therefore, a more specific question was whether this preference also lasts across several dots in a row. If so, we should observe a similar pattern for each sequential dot superimposed across participants. If not, we might observe a structured pattern for the first dot but a more widely distributed pattern for the subsequent dots. For the multiple-dots condition it was expected that, due to the persistent visibility of the placed dots, the effect of the hidden perceptual structure is generally reduced.

As a general method, the individual dots or dot patterns were superimposed across participants. It was then tested whether they could have been generated by a homogeneous spatial Poisson point process [44]. Such a process is characterized by two key properties: Homogeneity and independence. Homogeneity means that the points occur at each location with equal probability, while independence requires that the outcome in one region of space has no effect on the outcome in other regions.

To examine the sequential processes of location selection in detail, one method was to connect the subsequent dots of each participant by straight lines, as in the study of Gemelli and Alberoni [42]. These researchers observed that the individual trajectories formed broken lines with mostly acute angles. Here, the length and direction of the paths between subsequent dots were also analyzed.

As we will see, the participants were largely unable to randomly select locations. Rather, they were strongly affected by the distribution of regional attraction. Moreover, they followed, at least to some extent, a common sequential selection strategy.

## Experiment 1

The first experiment consisted of a single-dot condition (10S), in which one dot had to be placed ten times in a row at a random location within an empty square area. In view of the results reported in the literature, it was expected that the hidden perceptual structure of the empty area strongly affects the selection of location, i.e., that specific regions such as the center and the diagonals are salient and more attractive for placing dots than others. Accordingly, the dots should cluster in these regions. In addition, there was a multiple-dots condition (10M) that was similar to the single-dot condition, except that already placed dots remained visible. Because the visibility of the dots makes inhomogeneities more apparent, it was expected that the dots in condition 10M are more evenly distributed across the area than those in condition 10S.

### Method

#### Participants

Eighty-three persons (33 males, mean age 23.6, *SD* = 8.12), mostly students from all disciplines of the University of Konstanz, were recruited via a local online-system (ORSEE, Greiner, 2015) for the online experiment. As incentive, each participant had the chance to win one of eight vouchers worth 4 €. The study was performed in accordance with the ethical standards of the Declaration of Helsinki (1964) and was approved by the ethics committee of the University of Konstanz (Confirmation 27/2020). Participants were informed that they are free to withdraw from the study at any point in time without any negative consequences. Informed consent was obtained from all participants by check-marking a box on the informed-consent page before the actual experiment started.

#### Procedure

When the participants started the online experiment, they were asked to use a notebook or desktop computer. After confirming that this was the case, the actual experiment began with a short instruction that informed about the task and continued by requiring the participants to work through two conditions. Condition 10S consisted of ten trials. On each trial the participants had to place a single black dot (radius 5 px) at a randomly chosen location within a screen-centered empty white canvas (400 x 400 px) by clicking on the corresponding location with the computer mouse. The participants were instructed to imagine that the canvas is a sheet of paper on which a raindrop was falling on a random spot. There was no time limit. Participants could forward to the next trial by clicking on a corresponding button. In condition 10M, the task was also to randomly place 10 dots on the canvas, while the already placed dots remained visible. The order of conditions was randomized across participants. Altogether, the participants spent about 5 minutes on the study.

### Results

#### Spatial dot distribution

**Fig 1:**
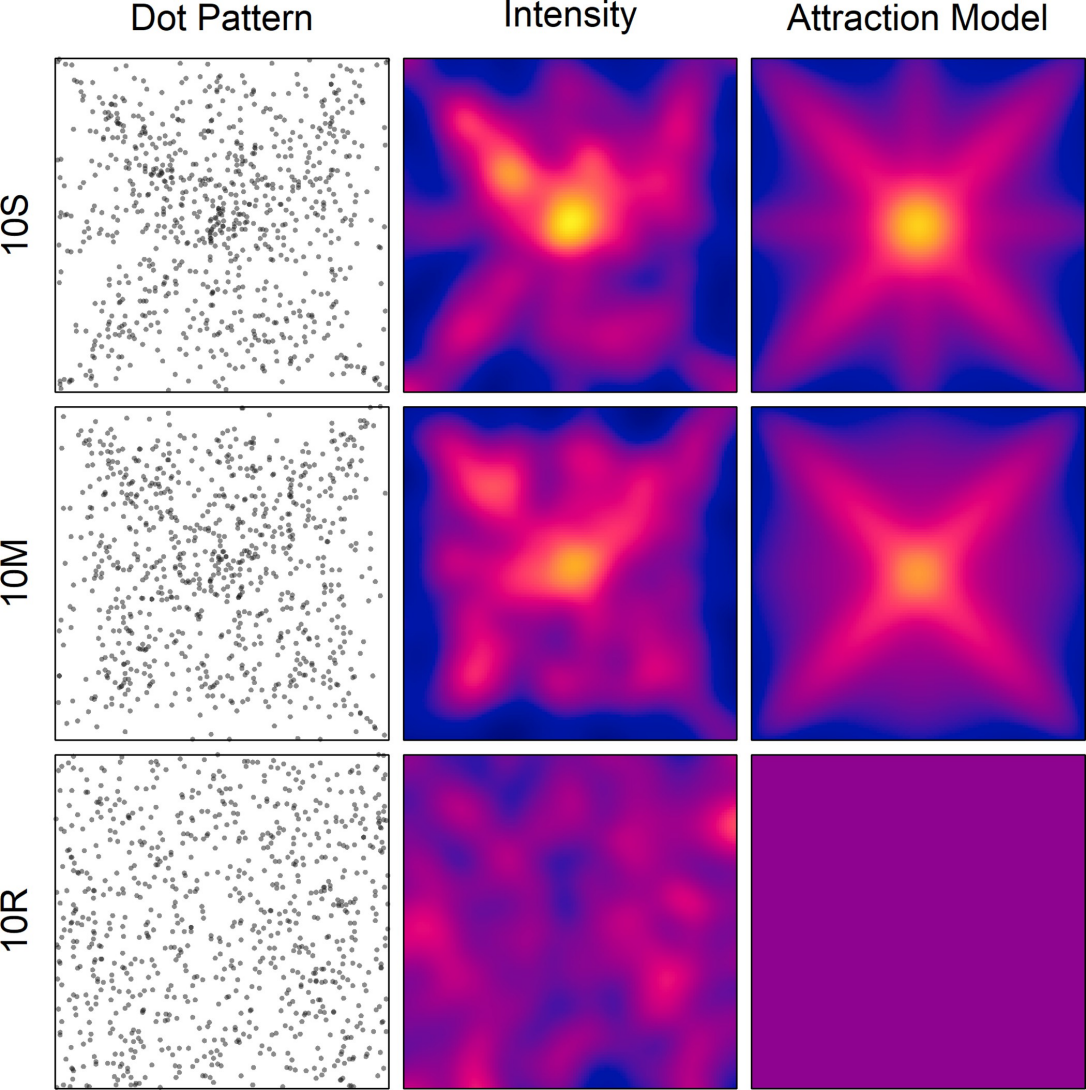
Superimposed dot patterns (column Dot Pattern) for the two conditions in Experiment 1, and for a random-dot pattern with the same number of dots (10R). The maps of the corresponding estimated intensities (column Intensity) are also shown. The intensity in the maps ranges from 0.0002 (blue) to 0.0122 (yellow). The right column depicts the maps of the modelled spatial attractions (see text for details).

The dot patterns for the two conditions, superimposed across participants, are shown in Fig 1. For comparison, a random-dot pattern (10R) is also shown. It was produced by uniformly distributing 830 dots over the area. As can be seen, the human-generated dots are not distributed homogeneously. Rather, they cluster along the principal axes, the diagonals, and at the center. As expected, this characteristic is more pronounced for condition 10S than for 10M. To test the attraction of the diagonals, the same procedure as in Lisanby and Lockhead [22] was applied. That is, the coordinates of the dots were first folded around the imaginary vertical axis and then around the horizontal axis that bisect the area, so that all dots are finally located in the lower left quadrant. If the diagonals attract the placement of dots, then there should be a positive correlation between the x and y coordinates. This was indeed the case. The correlation for condition 10S was *r* = 0.234, *t*(828) = 6.91, *p* < 0.001, and for 10M *r* = 0.197, *t*(828) = 5.78, *p* < 0.001. Moreover, testing across the individual correlations revealed that the mean correlations differed between the conditions (.199 vs. .142), *t*(82) = 1.34, *p* < .05 (one-sided). This indicates, as predicted, that the clustering along the diagonals was reduced in condition 10M.

A further result is that the participants placed more dots in the upper half of the area than in the lower one, which was the case for condition 10S (463 vs. 367), *t*(82) = 3.12, *p* < 0.01, as well as for 10M (451 vs 379), *t*(82) = 3.00, *p* < 0.01.

To make the underlying structure of the superimposed dot patterns more visible, the intensity functions of the corresponding point processes were estimated by means of the R [45] function “density.ppp” from the library “spatstat” [44]. The standard deviation (sigma) of the smoothing kernel was set to 20. Fig 1 shows the obtained intensity maps (column Intensity). The intensity reflects the average number of dots per unit area, which is one pixel (px) in the present case. If the participants had placed the dots in accordance with a homogeneous Poisson process, then the intensity should vary like that of the random-dot pattern (10R). This was obviously not the case. Rather, the intensity is concentrated along the diagonals, somewhat along the axes, but mostly at the center, while it is rather low in the outer regions.

To support this interpretation, the program “quadrat.test” [44] was applied to compute χ^2^ tests of *complete spatial randomness* (CSR). Such a test is based on quadrat counts, for which the area of a pattern is divided into equally sized cells. For the present analyses, a 5x5 grid was chosen. As result, the test was highly significant for both the 10S pattern, χ^2^(24) = 185, *p* < 0.001, and the 10M pattern, χ^2^(24) = 145, *p* < 0.001, indicating that they were probably not produced by a homogeneous Poisson process. In contrast, the test for the 10R pattern was not significant, χ^2^(24) = 21.3, *p* = 0.754.

#### Modeling regional attraction

The data and estimated intensities clearly show that the dots were not placed randomly across the area. Rather, specific regions were preferred. If one assumes that the intensity maps in Fig 1 roughly reflect the regional preferences, then they might be used to construct a formal model of regional attraction. A possible approach is demonstrated next. By following Arnheim [16, 17], it is assumed that the center, the diagonals, and the main axes are regions of high attraction. The attraction of each component, i.e., the horizontal axes, the vertical axes, the center, the major diagonal, and the minor diagonal, can then be represented by a corresponding bivariate normal distribution with density function *f*(x, y). For all functions, the means of the variables *X* and *Y* are fixed to the center of the area, i.e., to *μ*_x_ = *w*/2 and *μ*_y_ = *h*/2, where *w* denote the width of the area in px, and *h* its height. Because the shape of the density function is largely determined by the correlation between *X* and *Y*, it is convenient to define the covariance matrix in terms of the variances σ^2^_x_ and σ^2^_y_, and the correlation *ρ* between *X* and *Y*. Thus, we have 5x3 = 15 free parameters that must be determined for the five component distributions. To reduce their number, several simplifying assumptions are made by introducing three model parameters α, β, and γ. All density functions and how their free parameters are replaced by *h*, zero, or by one of the model parameters can be seen in Table 1.

**Table 1:** Density functions for the different regions and corresponding parameter values in terms of the three model parameters α, β, and γ, and the width (w) and height (h) of the area.

Regions	Function	Parameters				
		*μ*_x_	*μ*_y_	σ^2^_x_	σ^2^_y_	ρ
Horizonal axis	*f*_h_	w/2	h/2	α	h	0
Vertical axis	*f*_v_	w/2	h/2	h	α	0
Center	*f*_c_	w/2	h/2	β	β	0
Major diagonal	*f*_j_	w/2	h/2	α	Β	γ
Minor diagonal	*f*_i_	w/2	h/2	α	β	−γ

In a further step, the density functions are used for constructing three component maps of attraction for the area of 400x400 px, one map *M*_C_ = *f*_c_ for the center, one map *M*_A_ = *f*_h_ + *f*_v_ for the axes, and one map *M*_D_ = *f*_j_ + *f*_i_ for the diagonals. After scaling each component map so that its sum equals one, they are combined to a master map *M* of spatial attraction by multiple linear regression:

$$M=a_{0}+a_{D}M_{D}+a_{A}M_{A}+a_{C}M_{C.}$$ (1)

**Fig 2:**
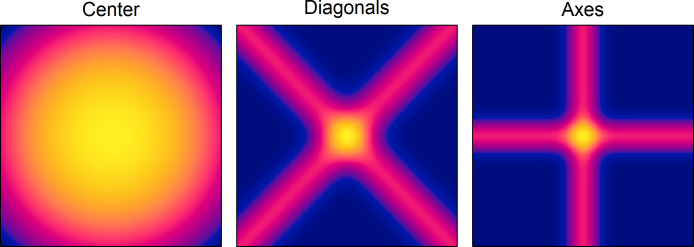
Estimated maps of attraction for the three regional components (center, diagonals, and axes) for condition 10S in Experiment 1. The range of values in the three maps are 6.0849x10^-5^ to 6.1131x10^-5^, 2.3756x10^-10^ to 2.7611x10^-4^, and 2.0420x10^-25^ to 4.8591x10^-04^, respectively.

Eq (1) is a formal model of spatial attraction in a rectangular area. This model was fitted to the data of condition 10S by means of the R program “optim” [45]. It searched for values of the three model parameters that maximize the correlation between the empirical intensity map and the master map of attraction. The obtained parameters are α = 7.933976x10^6^, β = 8.522916x10^6^, and γ = 0.999. The corresponding maps of attraction for the three regional components (Center, Diagonals, and Axes) can be seen in Fig 2.

The corresponding regression parameters are: *a*_0_ = −1.178, *a*_D_ = 26.8, *a*_A_ = 6.08, and *a*_C_ = 19334. The resulting master map of attraction can be seen Fig 1 (in column Attraction Model). Its correlation with the empirical intensity map is *r* = .867. It should be noted that the component maps also correlate. The correlation between the map of diagonals with that of the axes is *r* = .0559, that between the map of diagonals and the map of the center is *r* = .205, and that between the map of the axis and the map of the center is *r* = .515.

Applying the model to condition 10M revealed as model parameters α = 3.5612x10^4^, β = 4.39661x10^5^, and γ = 0.999. The regression coefficients are *a*_0_ = −31.9, *a*_D_ = 22.2, *a*_A_ = 0.638, and *a*_C_ = 5.22049x10^5^. The corresponding master map of attraction can also be seen in Fig 1. Its correlation with the empirical intensity map is *r* = .883.

For demonstrating the regional attraction for a random process, the corresponding master map is also shown in Fig 1. It is of course homogeneous.

#### Path lengths and directions

**Fig 3:**
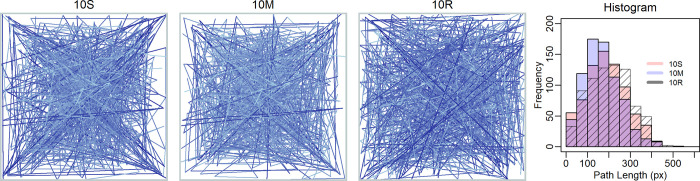
Line patterns resulting from connecting successive dots for each participant in the two conditions in Experiment 1, and for the random-dot pattern (10R). The blue gradient of the lines indicates their length: The longer the line the darker the blue. The corresponding histograms are shown in the outermost panel on the right.

Until now, we have analyzed and modeled the final dot patterns. To get an insight into the processes involved in the sequential selection of locations, the direct paths between successive dots were visualized by straight lines for each participant, as in Gemelli and Alberoni [42]. The corresponding patterns resulting from superimposing all lines across participants are shown in Fig 3. The blue gradient of the lines reflects their length. By considering the patterns, it becomes obvious that not only the dots were frequently placed near the diagonals, but also that the direct path between successive dots often run along or parallel to the diagonals, at least for condition 10S. Moreover, the perceived mean darkness of the line patterns indicates that the paths for condition 10S are longer than for 10M.

Fig 3 also shows the histograms of the path lengths. As can be seen, the distributions for both experimental conditions are positively skewed. However, the skewness is greater for condition 10M (skewness: .553) than for 10S (skewness: .258). Because the greater skewness is due to a higher proportion of shorter paths, the mean path length for condition 10M (169 pixels, *SD* = 84) is also significantly shorter than that for 10S (188 pixels, *SD* = 95), *t*(82) = 3.54, *p* < 0.001. Nevertheless, the skewness is smallest (.175) for the random-dot pattern. A computer simulation of 10^5^ random lengths within the square area revealed a skewness of .185. This demonstrates that random length is not distributed symmetrically in such an area.

**Fig 4:**
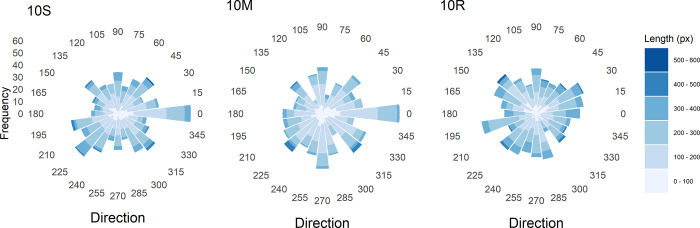
Histograms of direction (in polar coordinates) and length of the paths between successive dots for the two conditions (10S, 10M) in Experiment 1, and for the random-dot pattern (10R).

To also consider the distribution of path directions in combination with path length, the two measures were plotted analogous to a so-called *wind-rose diagram* (the used R plot function was a modified version of a function provided by Andy Clifton, see https://stackoverflow.com/users/2514568/andy-clifton), which is shown in Fig 4. If the directions were equally distributed, then 4.17% of the paths should occur in each of the 24 direction bins. However, a simulation with 10^5^ x 748 lines with random length and direction, categorized in the same way as the empirical ones, revealed that the directions are not distributed equally. Rather, the percentages vary systematically across bins from 4.06% to 4.34%. They are highest for the four diagonal directions (45°,135°,225°,315°). The corresponding percentages for the observed directions in condition 10S are 5.09%, 4.69%, 6.02%, and 5.22%. The simulated critical value for a significance level of .05 is 5.49 for all directions. Thus, only lines with a direction around 225° (down left) occurred significantly more frequent. For condition 10M the corresponding percentages are 5.09%, 4.28%, 5.09%, and 5.22%, of which none is significant.

If we consider the four axial directions (0°, 90°, 180°, 270°), then their simulated percentage in the corresponding bins is 4.24%, respectively. The observed ones for condition 10S are 8.03%, 4.55%, 2.95%, and 4.02%. Because the critical value is the same as for the diagonal directions, we can conclude that only the directions in the 0° (to right) bin occurred significantly more frequent. For condition 10M the percentages are 8.03%, 4.82%, 4.15%, and 5.76%. Thus, directions close to 0° were again significantly more frequent, but also those close to 270° (down).

Concerning the lengths, it can again be seen that in condition 10M more paths are of short or medium length, compared to 10S. Moreover, most of the longest paths in condition 10M have a direction close to the diagonal directions of 225° or 315°.

#### Sequence of locations

**Fig 5:**
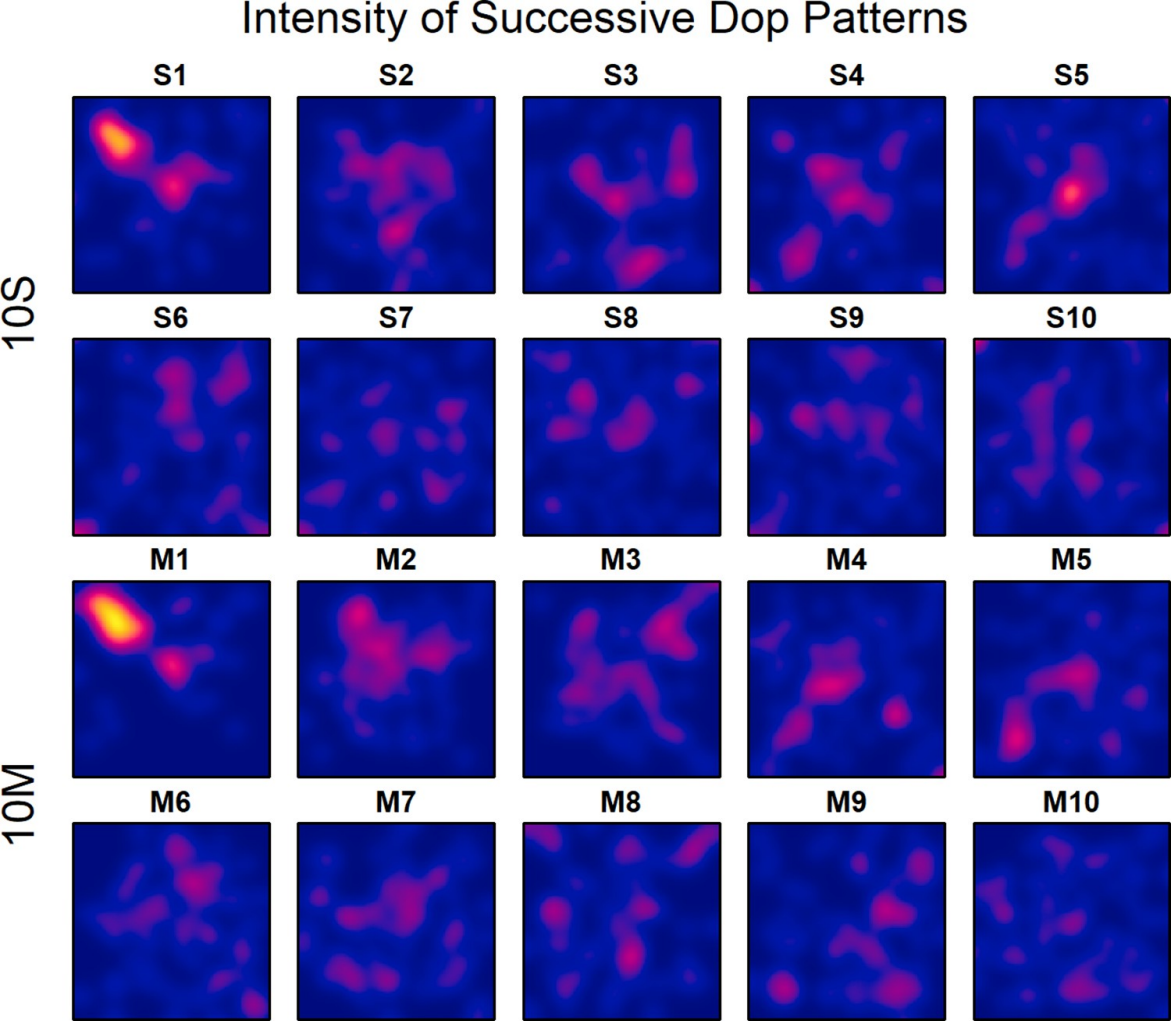
Intensity plots of the superimposed dot patterns for the ten successive location selections in the two experimental conditions in Experiment 1.

To extend the analyses of the sequential selection process of locations beyond that of pairs of consecutive dots, the intensities were also plotted separately for each of the ten selections in the two experimental conditions. As can be seen in Fig 5, most participants placed the first dot near the minor diagonal in the top-left quadrant. The second dot was then placed more towards the center. This sequence holds for both conditions, which indicates that, at least at the beginning, most participants followed a common temporal pattern to place the dots. Not surprisingly, at later steps the selected locations largely differ between participants.

**Fig 6:**
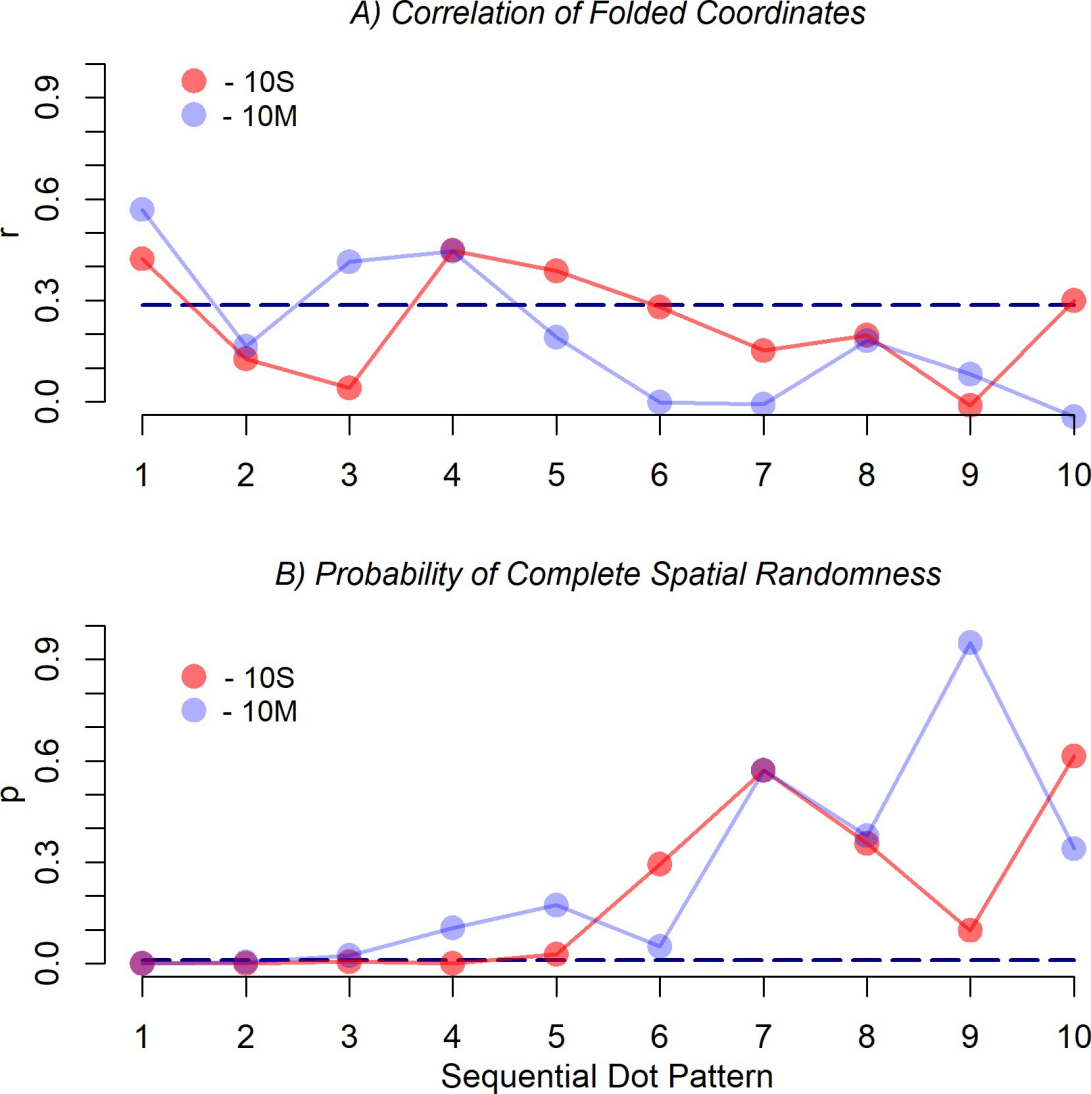
Correlations of the two-times folded coordinates (panel *A*) and *p*-values of the quadrat (CSR) tests (panel *B*) for the successive ten superimposed dot patterns in the two experimental conditions in Experiment 1. The dashed line in the upper panel indicates the critical value for a significance level of .05, i.e., values above this line are significant at this level. In the lower panel the dashed line indicates *p* = .01.

For analyzing how persistently the dots were placed near the diagonals, the correlations of the two-times folded coordinates of the dots were computed separately for each of the ten sequential superimposed dot patterns. The result is shown in panel *A* of Fig 6. As can be seen, the correlation for the first dot is relatively high for both conditions (10S: *r* = .423; 10M: *r* = .569). For the next dot it is considerably lower. Later, the correlation rises and falls again. In condition 10S the maximum correlation (.448) was reached at dot 4. Overall, it seems that the correlation does not vary randomly across the sequence of dots but rise and fall like a damped wave line, where the wavelength is slightly larger for condition 10S.

Inspecting correlations of the dot coordinates is rather selective because they only detect the preference of the diagonals, and only if the individually preferred location near the diagonals varies to some extent across participants for a given dot in the sequence. If participants commonly prefer the center or a specific corner, then the correlation remains relatively small. Therefore, to examine to what extent the dots have generally been placed at commonly preferred locations in the sequence, a CSR test was computed for each of the sequential dot patterns. The dots in each pattern were summarized by the nine quadrats of a 3×3 grid. Given the relatively small number of dots in the sequential patterns, a finer grid was not applicable. As mentioned, the CSR test provides the probability *p* that the pattern is compatible with a homogeneous Poisson process. The obtained *p* values for the two experimental conditions are shown in panel *B* of Fig 6. Even if we choose a significance level of *p* = .01, it can be concluded for condition 10S that the participants placed the first four dots in locations also preferred by many of the other participants (*p* for dot number 5 is .027). Only later they selected less commonly preferred locations. In condition 10M, the desynchronization already started after the first two dots (*p* for dot number 3 is .023).

### Discussion

In this experiment the participants were instructed to simulate rain by randomly placing 10 dots in a white square area on the screen. In a single-dot condition each dot had to be placed in an empty square area, whereas in a multiple-dots condition the already placed dots remained visible. The general question was how the resulting dots are distributed in the different conditions. From the literature it is already known that location selection is biased by the hidden structure of the area [22, 41]. For a square area this means that the regions near the diagonals, axes, and center are highly attractive for being selected. This attraction was also observed in the present experiment. In addition to placing more dots in the upper half of the area than in the lower one, the participants placed dots frequently in the mentioned regions, while outer zones were largely avoided (see Fig 1). As expected, the regional attractions were more pronounced in the multiple-dots conditions, i.e., when the already placed dots remained visible.

The preference for the diagonals was also confirmed by the significant correlations of the two-times folded coordinates of the dots. Compared to those reported in Lisanby and Lockhead [22], though, they were much smaller, even in the single-dot condition (0.23 vs. 0.77). However, the 195 participants in that study had to place only one single dot. If we consider only the pattern for the first dot in the present experiment, then the correlation increases to .42 in condition 10S, and even to .57 in 10M. Possible reasons for why these correlations are higher than the average ones will be discussed below. At the moment it is important to note that the dot patterns for both experimental conditions deviate significantly from complete spatial randomness, i.e., from patterns generated by a homogeneous spatial Poisson process. An example of a pattern (10R) that does not significantly deviate is shown in the last row of Fig 1.

The underlying structure of the dot patterns is roughly reflected by the intensity maps (column Intensity in Fig 1). Obviously, the highest intensity is at the center, especially for the single-dot condition. Moreover, for this condition there is also an additional large peak on the minor diagonal. Interestingly, the two highest peaks correspond to the positions C3 and B4 in Falk, Falk [19], respectively, which were also marked most frequently in their 5×5 grid. As expected, the regional bias is somewhat reduced when previously placed dots remain visible.

Thus, the overall results support the notion that people are largely biased in selecting random locations within an area, especially when there are no other visible dots. In the modeling section it has been shown how the regional attraction of the center, the diagonals, and the axes might be recovered from the intensity maps of the different conditions. The obtained master maps of regional attraction are shown in Fig 1 (column Attraction Model). By considering the parameter values of the individual component maps (see Fig 2) it is obvious that the center map plays a major role. Interestingly, different from what one might have expected, the center map is less responsible for the high peak of attraction at the center, but more for the attraction at some distance to the center that is neither explained by the attraction of the diagonals nor by that of the axes. This especially holds for the multiple-dots condition. It should be noted, though, that the relation between the regression coefficients should be interpreted with caution. The large value for the center map of attraction is mainly due to the broad distribution. The applied scaling, so that its overall attraction sums up to one, leads to rather small values, which then require a large amplification by the corresponding regression coefficient.

A further result is that not only the dot patterns of the two conditions differ, but also the planning and realization of the dot sequences. This is shown by analyzing the sequential selections. When pairs of successive dots were connect by lines for each participant, as in Gemelli and Alberoni [42] and the superimposed lines of all participants are considered (Fig 3), then it is obvious that there are more shorter lines for the multiple-dots condition. As a consequence, the corresponding histogram of path length is more positively skewed, compared to that for the single-dot condition. Usually, the distribution of random line lengths approximates a symmetric triangular distribution [46]. However, given the restrictions of a square area, where longer paths are possible between the corners, this is not the case. A corresponding computer simulation revealed that for the present case the distribution of the random path length is positively skewed.

By considering Fig 3 it can also be seen that not only the dots were placed near the diagonals, but that the paths between successive dots also often run along them, or at least in parallel. How the path directions are distributed in each condition can be seen in the ‘wind-rose’ diagrams (Fig 4). For the single-dot condition there was a high percentage of paths directed around 225° (down left) and 0° (to right). In contrast, for the multiple-dots condition, frequent paths occurred only in bins around two axial directions 0° and 270 (down). Thus, disproportionately many paths with a diagonal direction occurred merely in the single-dot condition, whereas paths directed from left to right occurred frequently in both conditions.

The differences in path length and direction further indicate that the sequential selection process of locations differed between the two conditions. They suggest that in the single-dot condition the participants tried to simulate a spatial random process, whereas in the multiple-dots condition a spatial random pattern of dots was planned for which the realization of a *random sequence* of locations played a subordinate role. It should be noted that, different from Ross and Weiner [34], the participants were not explicitly instructed to also select a random *sequence* of locations. However, it is likely that they, at least for the single-dot condition, implicitly assumed that locations of subsequent dots should be independent.

In addition to the pairwise spatial relation between successive dots, the superimposed dot patterns were also analyzed separately for each sequential dot. To examine how persistently the dots were placed near the diagonals, the correlations of the two-times folded coordinates of the dots were computed for each of the ten dots. As can be seen in panel *A* of Fig 6, for both conditions the correlation for the first dot is significantly high, but then it dops below significance for the second dot. Overall, it seems that the correlations rise and fall across the dot sequence like a damped wave, which indicates a negative recency effect. The participants first selected a location near a diagonal, but next some location off the diagonals. Later dots were again placed near the diagonal and so on.

A surprising result is that the first dot was commonly placed in a rather restricted area. If we consider Fig 5, then it is obvious that most participants placed the first dot along the minor diagonal in the upper-left quadrant, which holds for both experimental conditions. Thus, whereas the overall pattern, in particular that for the single-dot condition, looks similar to that observed in other studies where only one single dot had to be placed, this is not the case for the patterns of the first dot alone. This indicates that the number of dots to be placed strongly influenced the location of the first dot. The fact that the participants largely agreed to start near the minor diagonal in the left upper quadrant, indicates that the sequence of dot locations was planned in advance, at least to some extent.

To examine whether this performance depended on the order of the conditions, which was randomized, both orders were also analyzed separately. Of the 35 participants who started with the single-dot condition, 20 (57%) placed their first dot in the upper left quadrant in that condition, and 21 (60%) did this in the subsequent multiple-dots condition. Of the 48 participants who started with multiple-dots condition, 35 (73%) placed the first dot in the upper left quadrant in that condition, and 23 (48%) did this in the subsequent single-dot condition. This demonstrates that in both conditions there was a strong tendency to place the first dot near the minor diagonal in the upper left quadrant irrespective of the condition and their order.

The fact that the correlation of the two-times folded coordinates of the dots was nevertheless relatively high already for the first dot, although only a small region of the diagonals was used, shows that a high correlation has to be interpreted with care. Placing many dots along one half of one diagonal already leads to the same high correlation as when the dots were evenly scattered along both diagonals. Folding the dot coordinates two times makes the difference in performance disappear. Thus, a high correlation does not guarantee that the dot pattern looks like an oblique cross.

The correlation measure is also unsuitable for detecting preferred regions other than those along the diagonals, like the axes or the center. Thus, an absent correlation in a two times folded dot pattern does not imply the absence of a commonly selected region. To examine for each sequential dot patten whether such regions were present, it was tested to what extent the patterns are compatible with a homogenous Poisson process. The *p*-values for the intensities in Fig 5 are shown in panel *B* of Fig 6. As can be seen, in the single-dot condition the participants placed at least the first four dots in locations also preferred by other participants. Only then they selected less commonly preferred locations. In the multiple-dots condition desynchronization already started after the first two dots. This indicates that the first dots were systematically placed at commonly preferred locations, although not necessarily at the diagonals. Only then less commonly preferred locations were selected. In the multiple-dots condition, less common locations were already selected somewhat earlier, which demonstrates that the visibility not only affected the overall patterns but also the strategies of location selection.

## Experiment 2

After basic results on position biases in simple selection conditions were obtained in Experiment 1, this experiment served for examining two further issues. First, in an extended multiple-dots condition the effects of an increased number of dots, compared to Experiment 1, should be examined. As we have seen, if ten dots have to be placed, they cluster in central regions, while outer areas are largely ignored. This might change with more dots. As mentioned, Ross and Weiner [34] required to mark 64 cells in a 20×20 grid and observed rather regular patterns. Similar results were observed for the free placement of 20 dots by Gemelli and Alberoni [42]. In the present experiment the participants had to place 30 dots in a multiple-dots (30M) condition. It was expected that the dots are now more evenly distributed across the whole area. This does not necessarily mean that the patterns are compatible with a homogeneous Poisson process, because they might be too regular now. As can be seen in Fig 1 (10R), a homogeneous Poisson process does produce clusters and empty spaces, but unsystematically.

The second aim of this experiment was primarily methodological in scope. In Experiment 1, the randomness of the superimposed dot patterns was assessed by testing for complete spatial randomness, i.e., whether they are compatible with a homogeneous spatial Poisson process. However, by doing this, an important property of the Poisson process was ignored. Such a process does not only produce dots at random locations, but also a random number of dots, which follows from its two key properties mentioned in the Introduction, homogeneity and independence [44]. Thus, by requiring the placement of a fixed number of dots in an area, the latter assumption was violated. Furthermore, the violation also holds for the random-dot pattern in Fig 1 (10R). By producing a fixed number of uniformly distributed dots, a binomial point process was simulated rather than a Poisson point process [44]. This raises the question whether the violation of independence might lead to invalid conclusions in the present study. The fact that the CSR test was not significant for the random pattern suggests that it does not. Nevertheless, this issue should be tested more directly in the present experiment. Therefore, a specific multiple-dots condition (XM) was included, where the participants were free to place as many dots as the wanted. This allowed them to simulate a spatial Poisson process. Moreover, because the superposition of independent Poisson processes is also a Poisson process, it is now possible that the superimposed dot pattern is indeed compatible with a spatial Poisson process. Accordingly, it is justified to assess its homogeneity with the CSR test.

For condition XM it was also of interest to see over which range the number of produced dots would extend. To prevent any systematic influence on that range, all participants started with that condition.

### Method

The procedure was similar as in the multiple-dots condition in the previous experiment. There were two such conditions. In the first condition (XM), participants were instructed to simulate rain and randomly mark as many locations of rain drops as they wanted by placing black dots on the canvas. In a subsequent condition (30M), they had to place 30 dots at random locations. The order of the two conditions was fixed. The sixty-three persons (19 males, mean age 21.7, *SD* = 2.49) who participated were students from all disciplines of the University of Konstanz. None of them participated in Experiment 1. For taking part in the present online experiment, each participant had the chance of winning one of six 5€ vouchers.

### Results

The number of dots produced in condition XM ranged from 1 to 158 (Mean 29, SD 28). Interestingly, they sum up across participants to 1832, which is close to the fixed number of 1890 dots in condition 30M.

#### Spatial dot distribution

**Fig 7:**
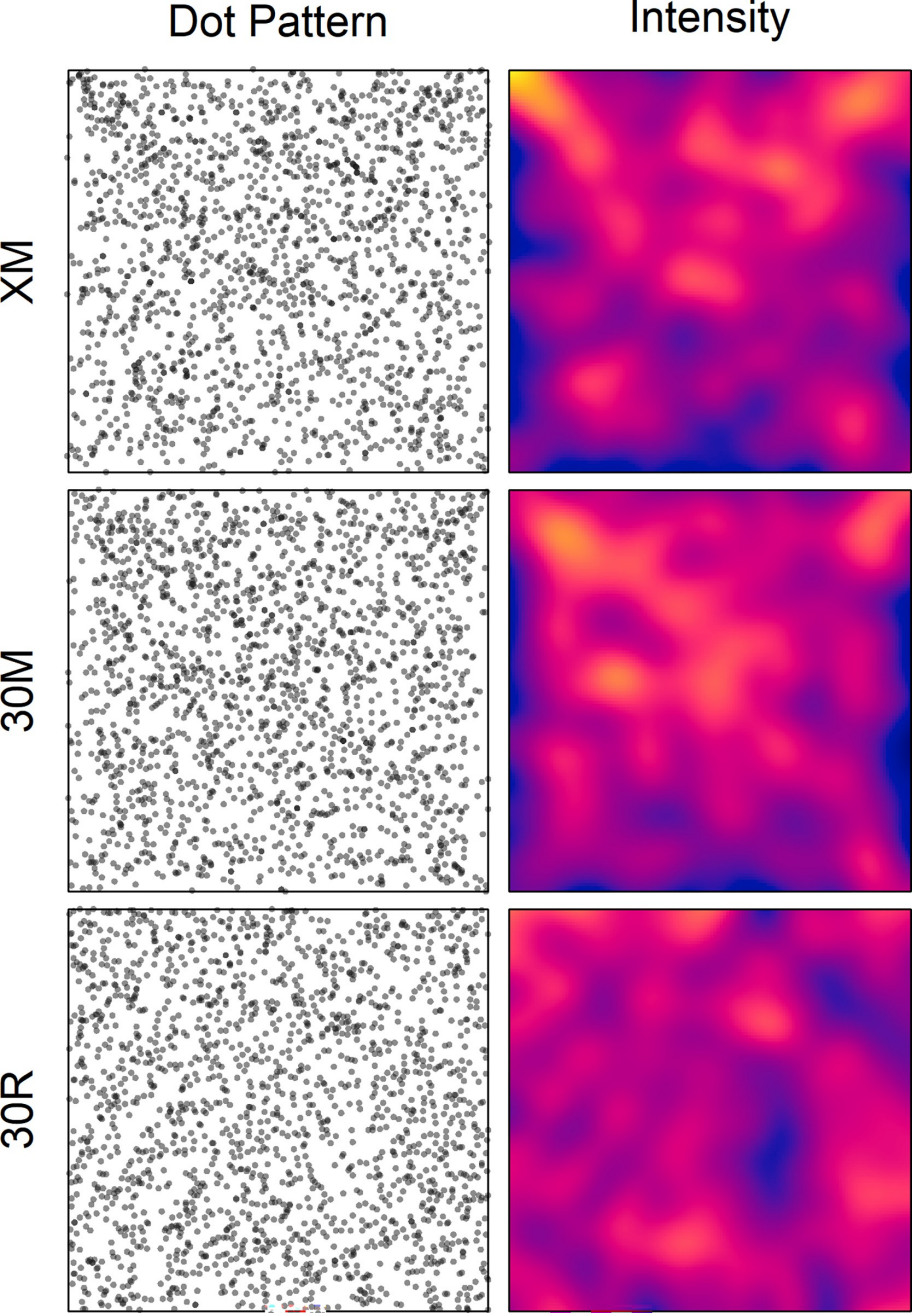
Patterns (column Dot Pattern) of superimposed individual dot patterns and corresponding intensity plots (column Intensity) for the two conditions in Experiment 2, and for a corresponding random-dot pattern (30R). The density ranges from 0.0048 (blue) to 0.0233 (yellow).

The superimposed dot patterns and the corresponding intensity plots for the two conditions are shown in Fig 7 (column Dot Pattern). For comparison, a random-dot pattern of 1890 dots (30R) and its intensity are also provided. As can be seen, the results are rather similar to those in Experiment 1. The center, diagonals, and axes were favored, while outer regions were avoided, even though to a lesser extent. Accordingly, the two-times folded dot coordinates correlated significantly for condition XM, *r* = .111, *p* < .001, as well as for condition 30M, *r* = .097, *p* < .001. Furthermore, the participants again placed more dots in the upper half of the area than in the lower part (XM: 999 vs. 833, *t*(61) = 2.25, *p* < .05; 30M: 1028 vs. 862, *t*(61) = 3.29, *p* < .01).

Because of the large number of dots, χ ^2^ tests could now be computed with a 10x10 grid. They revealed highly significant deviations from *complete spatial randomness* for the XM pattern, χ^2^(99) = 251, *p* < .001, as well as for the 30M one, χ^2^(99) = 245, *p* < .001, indicating that the patterns do probably not result from a homogeneous spatial Poisson process. For the random pattern we have χ^2^(99) = 97.8, *p* = .971.

**Fig 8:**
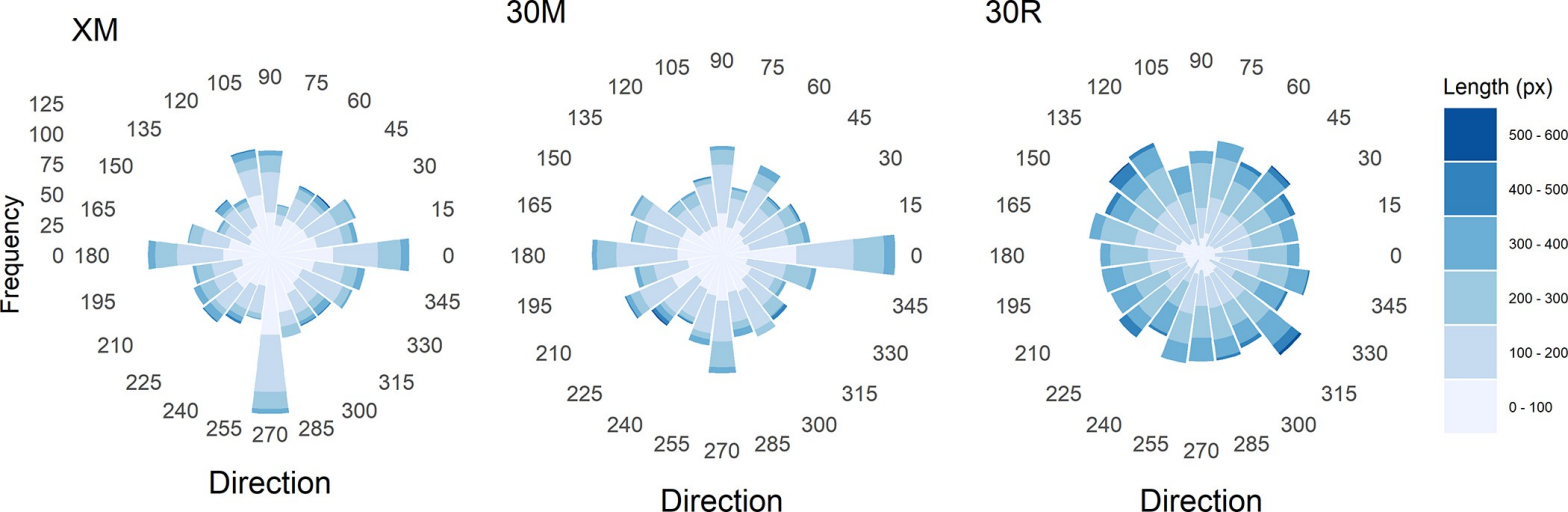
Histograms of direction (in polar coordinates) and length of the paths between successive dots for the different conditions in Experiment 2, and for the random-dot pattern (30R).

The mean path length between subsequent dots was significantly shorter in condition XM than in 30M, (134 px vs. 152 px), *t*(61) = 2.56, *p* < .05, (the participant with only one dot in XM was excluded from this analysis). Moreover, for both conditions the mean path lengths were shorter than that for the random-dot pattern (211 px). The differences are impressively visualized by the ‘wind-rose’ diagrams (Fig 8).

The ‘wind-rose’ diagrams also show that for both experimental conditions paths close to the diagonal directions are not disproportionately frequent. In contrast, paths near one of the axial directions (0°, 90°, 180°, 270°) are relatively frequent. For the XM condition the percentages are 6.63%, 4.96%, 5.82%, and 7.55%. Thus, according to the critical percentage simulated in Experiment 1, all percentages are significant, except for the 90° direction (up). The percentages for condition 30M are 8.18%, 4.87%, 5.86%, and 5.31%, indicating that path close to the two horizontal directions 0° and 180° (to right, and to left) are significantly more frequent.

### Discussion

In the first condition (XM) of this experiment, the participants were allowed to randomly place as many dots as they wanted. As a result, their number ranged from 1 to 158. In the second condition (30M), the participants had to randomly place 30 dots. In both conditions previously generated dots remained visible. Interestingly, despite the large range of dots in condition XM, summed up across the 63 participants, their number is similar to that in 30M. Thus, the overall number of dots placed by each participant in 30M was exactly three times larger, and, on average, almost three times larger in XM compared to condition 10M in Experiment 1. Nevertheless, if we consider the patterns in Fig 7, we see that in both conditions the dots, despite their larger number, still cluster along the diagonals, at least to some extent, and rarely occur in the outer regions, except the corners. This biased location selection is also confirmed by the significant correlations between the two-times folded dot coordinates, although they are smaller, compared to the previous experiment. Moreover, both dot patterns also deviate significantly from those of a homogeneous Poisson process. Importantly, the test for homogeneity was quite similar for both conditions, suggesting that a fixed number of dots is not problematic for the present objective.

A further result is that the average path length is greater for condition 30M than for XM. Furthermore, in both conditions it is shorter than that for the random-dot pattern. The analyses of the path directions revealed that, as in Experiment 1, diagonal directions did not occur disproportionately often. However, in both conditions horizontal directions occurred relatively frequently. In condition XM, there were also many paths with a direction close to 270° (downwards).

Taken together, the results show that even subjective random patterns of up to 30 dots are incompatible with a homogeneous Poisson process. This is due to specific regional preferences rather than to a too regular distribution across the whole area.

## Experiment 3

The two previous experiments demonstrate that certain regions in an area are differently attractive for being selected to randomly place a dot. Most attractive were the center, the diagonals, and the axes. However, the results also show that, if several dots have to be placed consecutively, the degree to which certain regions are actually selected depends on the sequential position, and on whether previously placed dots remain visible or not. The aim of this experiment was to generalize the results to a rectangular form of the area.

Accordingly, the experiment was similar to Experiment 1, except that the area was horizontally extended by one third. Thus, the area was not only rectangular, but also larger. In view of the results in the first two experiment, one might expect that the diagonals are also attractive in a rectangular area. Former studies, however, show that this is not necessarily the case. Psotka [41], for instance, observed that the dots in a rectangular area did not cluster along the diagonals but along a skeleton or stick figure [47] that looks like the inward pointing half of the Müller-Lyer illusion [48], i.e., along a short horizontal line in the center with arrowheads at either end pointing toward each other. Later, Lisanby and Lockhead [22] observed similar results. It should be noted, however, that these patterns were obtained by asking participants to place one single dot only. Thus, the question was whether a similar pattern also occurs for sequences of dots, and if so, already for the first dot, or only for the overall pattern.

### Method

Eighty-one persons (17 males, mean age 24.1, *SD* = 7.78), mostly students from all disciplines of the University of Konstanz, were recruited for participating in the online experiment. None of them participated in Experiment 1 or 2. As incentive, each participant had the chance of winning one of six vouchers worth 4€. The procedure was similar as in Experiment 1, except that a rectangular area of 600x400 px was used as canvas.

### Results

#### Spatial dot distribution

**Fig 9:**
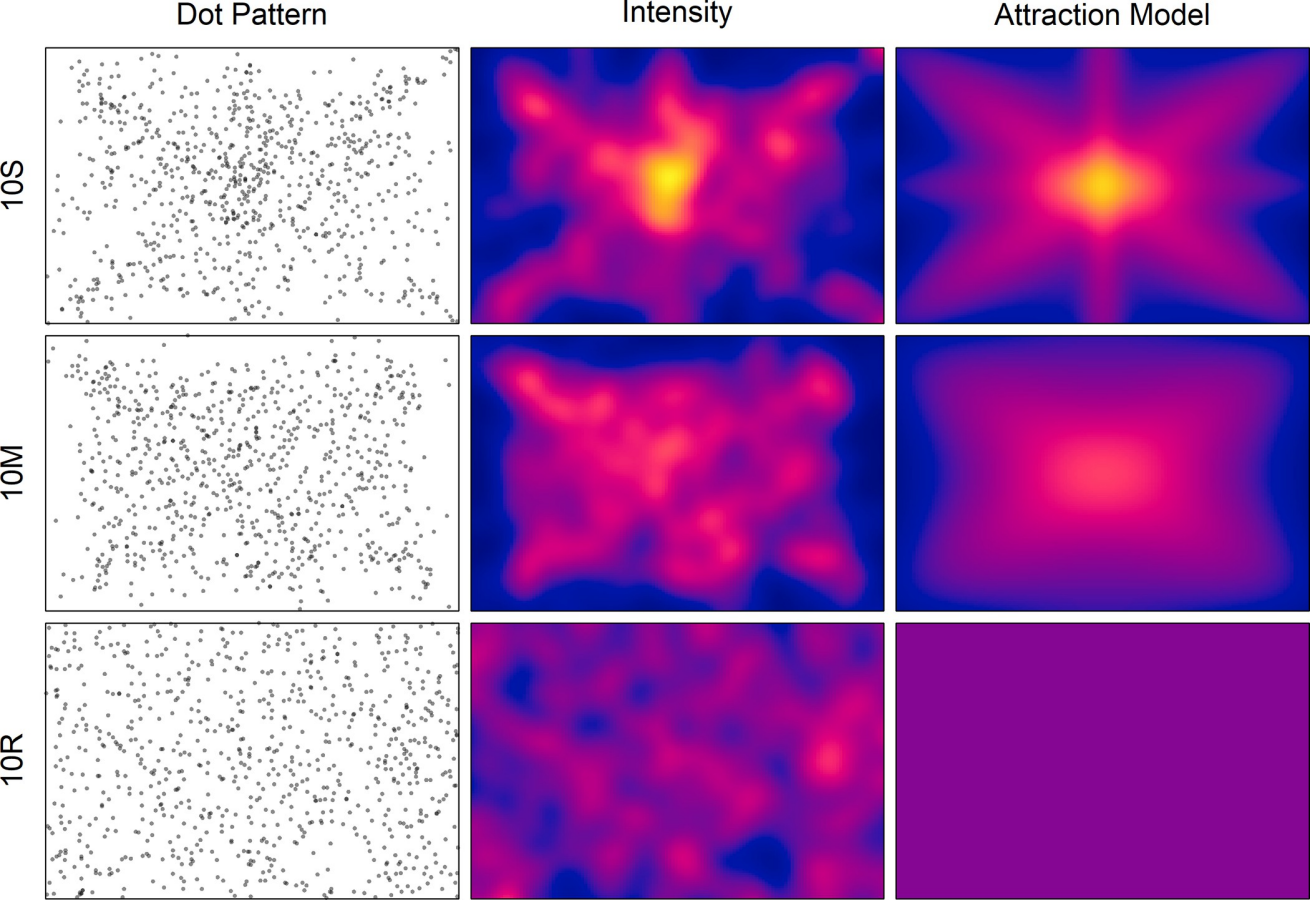
Superimposed dot patterns (column Dot Pattern) for the two conditions in Experiment 3, and for a corresponding random-dot pattern (10R). The maps of the corresponding estimated intensities (column Intensity) are also shown. The intensity in the maps ranges from 0.00 (blue) to 0.013 (yellow). In the right column the corresponding maps of the modelled regional attractions are depicted (see text for details).

The superimposed dots for each of the two conditions are shown in Fig 9 (column Dot Pattern). For comparison, a random-dot pattern (10R) with the same number of dots as in the two conditions is also shown. As can be seen, the dots are not distributed homogeneously. Rather, they cluster at the center and along the diagonals and the principal axes. Moreover, in condition 10S clustering is generally stronger, especially in the center. In condition 10S there was again a tendency to place dots along the vertical axis. Correlating the two-times folded coordinates revealed a correlation of *r* = .272, *t*(808) = 8.03, *p* < .001, for condition 10S, and of *r* = .159, *t*(808) = 4.58, *p* < .001, for condition 10M. Testing across the correlations of the individual participants revealed a significant difference between the corresponding means (.207 and .105), *t*(80) = 2.03, *p* < .05.

As before, participants placed more dots in the upper half of the area than in the lower half. However, the difference was significant only for condition 10S (443 vs. 367), *t*(80) = 2.67, *p* < .05, but not for 10M (424 vs. 386), *t*(80) = 1.51, *p* = .135.

The intensity maps of the dot patterns are also shown in Fig 9 (column Intensity). Obviously, the intensity is generally low in the outer regions. Moreover, for condition 10S it is more concentrated along the diagonals and much higher in the center, compared to 10M. Nevertheless, CSR tests with a 7x5 grid revealed a significant deviation from a homogeneous Poisson for condition 10S, χ^2^(34) = 285, *p* < .001, as well as for 10M, χ^2^(34) = 603, *p* < .001. For the random-dot pattern (10R) the χ^2^ test was not significant, χ^2^(34) = 37.3, *p* = .636.

#### Modeling regional attraction

Maps of regional attraction were constructed in the same way as in Experiment 1. The obtained parameter values for condition 10S are α = 1.0669x10^4^, β = 6.705x10^3^, and γ = 0.999, and the values of the regression parameters are: *a*_0_ = −0.152, *a*_D_ = 24.7, *a*_A_ = 5.53, and *a*_C_ = 2523. The resulting master map of attraction can be seen in Fig 9 (in column Attraction Model). Its correlation with the empirical intensity map is *r* = .863.

For all model fits there was the constraint that the regression coefficients for the component maps of attraction must be greater than zero. Here, this was relevant for condition 10M, where the coefficient of the component map for the axes (*M*_A_) became negative. Therefore, the model included only the component maps for the diagonals (*M*_D_) and the center (*M*_C_). The model parameter values are α = 2.1010x10^4^, β = 1.0861x10^4^, and γ = 0.999, while the regression parameters are: *a*_0_ = −0.309, *a*_D_ = 23.6, and *a*_C_ = 5089. The resulting master map of attraction is shown in Fig 9. Its correlation with the empirical intensity map is *r* = .809.

#### Path lengths and directions

**Fig 10:**
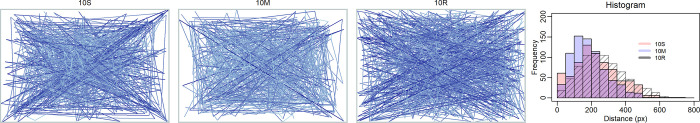
Line patterns resulting from connecting successive dots for each participant in the two conditions in Experiment 3, and for the random-dot pattern (10R). The blue gradient indicates the length of the lines: The longer the line the darker the blue. The corresponding histograms are shown in the outermost panel on the right.

The patterns of the superimposed paths between successive dots are shown in Fig 10. An analysis revealed that the mean length was 231 px (*SD* = 129) for condition 10S, 190 px (*SD* = 104) for condition 10M, and 266 px (*SD* = 129) for the random-dot pattern. A comparison of the length across participants revealed a significant difference between the two experimental conditions, *t*(80) = 4.37, *p* < .001. That the lengths differ can also be seen in Fig 10, where the average blue is larger for the lines in condition 10S.

The fact that the mean path length is smaller in condition 10M results from a higher proportion of shorter paths. This can be seen in Fig 10, where the histograms for the lengths in the different conditions are also shown. The distributions for all conditions are positively skewed. However, the skewness for condition 10M is larger than that for 10S (.908 vs. .476). The skewness for the distribution of the path lengths between the random dots is .438.

**Fig 11:**
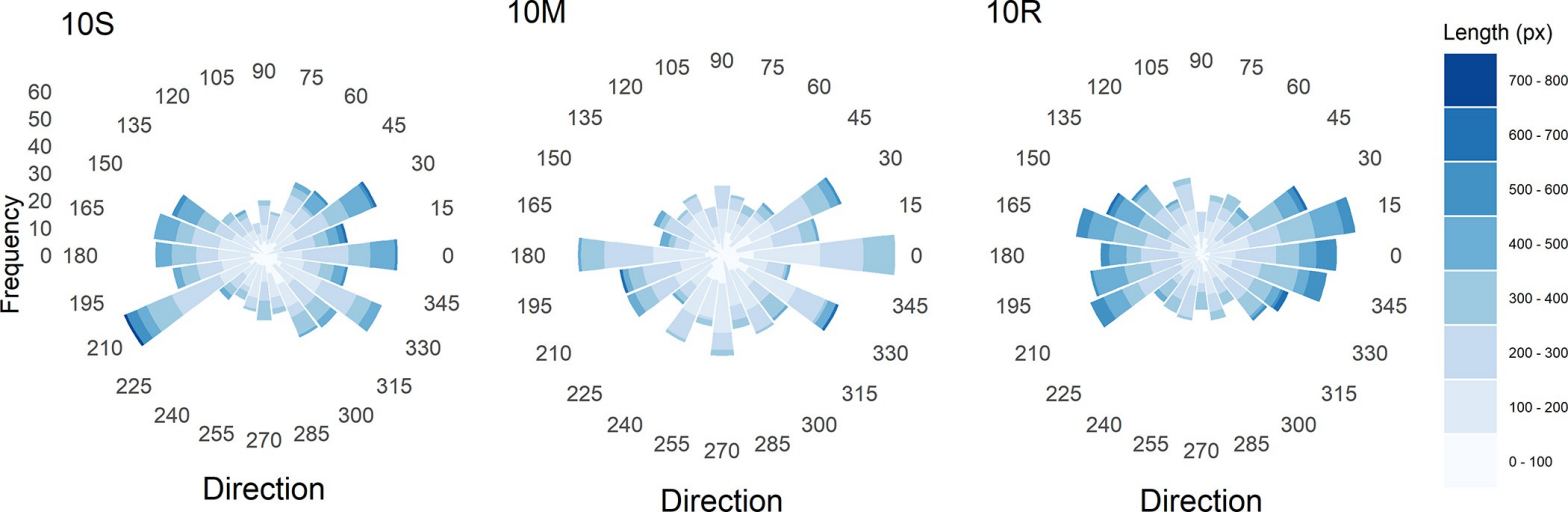
Histograms of direction (in polar coordinates) and length of the paths between successive dots for the two conditions (10S, 10M) in Experiment 3, and for the random-dot pattern (10R).

The ‘wind-rose’ diagram of the path directions and lengths are shown in Fig 11. It should be noted that, because the diagonals in a rectangle are not perpendicular to each other, their directions (33.7°, 146°, 214°, 326°) are different from those for a square area. Accordingly, to examine whether the diagonal directions occurred more frequently, we now consider the bins around 30°, 150°, 210°, 330°. For condition 10S the percentages in these four bins are 6.17%, 5.08%, 7.68%, and 6.45%. A computer simulation analogous to that in Experiment 1 revealed a common critical percentage of 6.17% (*p* < .05) for the four diagonal directions. Thus, for the directions down left and down right, the proportions of paths are significantly increased. Of the four percentages for condition 10M (6.17%, 3.57%, 4.94%, 6.036%) none is significant.

The critical percentage for the axial directions (0°, 90°, 180°, 270°) differs between horizontal and vertical (7.68% vs. 3.84%). Applying these values reveals that none of the four percentages for condition 10S (6.72%, 2.74%, 5.49%, 3.29%) is significant. For condition 10M, the corresponding percentages are 8.92%, 3.29%, 6.86%, and 4.80%. Thus, the direction from left to right is significant, as well as the down-right direction (270°).

With respect to the path lengths, it can again be seen in Fig 11 that, on average, they are shorter for condition 10M than for 10S. Not surprisingly, the longest lengths (darkest blue) have paths with a direction close to that of the diagonals.

#### Sequence of locations

**Fig 12:**
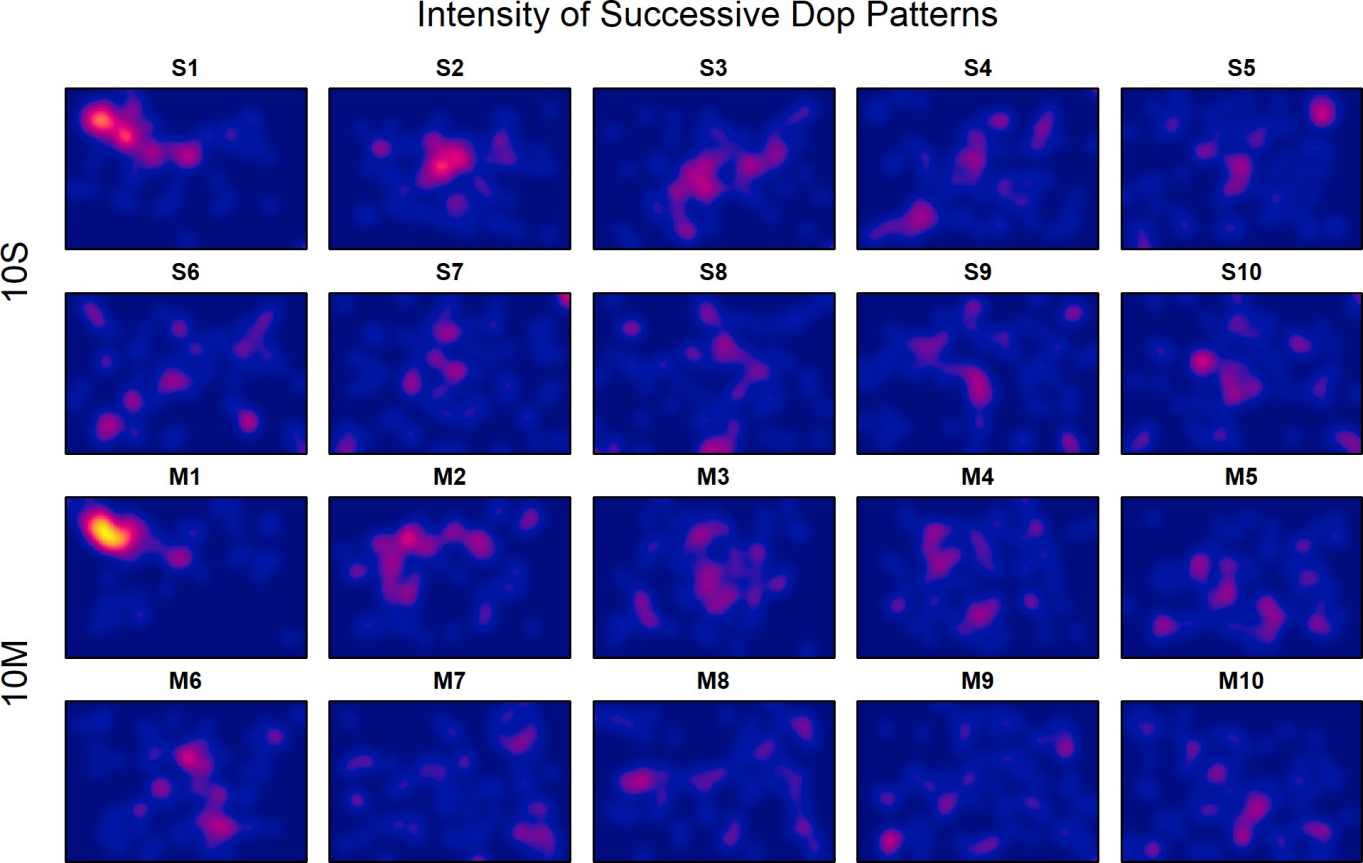
Experiment 3: Intensity plots of the ten successive dot patterns superposed across participants for conditions 10S and 10M, respectively.

The intensity plots for each of the ten successive dots in each condition are shown in Fig 12 for the two conditions. As can be seen, in the first step many participants placed their dot along the minor diagonal in the top-left quadrant and the next one more towards the center. This order is similar for both conditions and indicates that many participants started, as in Experiment 1, with a common sequence of locations to place the dots. Later, the selected locations largely diverged between the participants.

**Fig 13:**
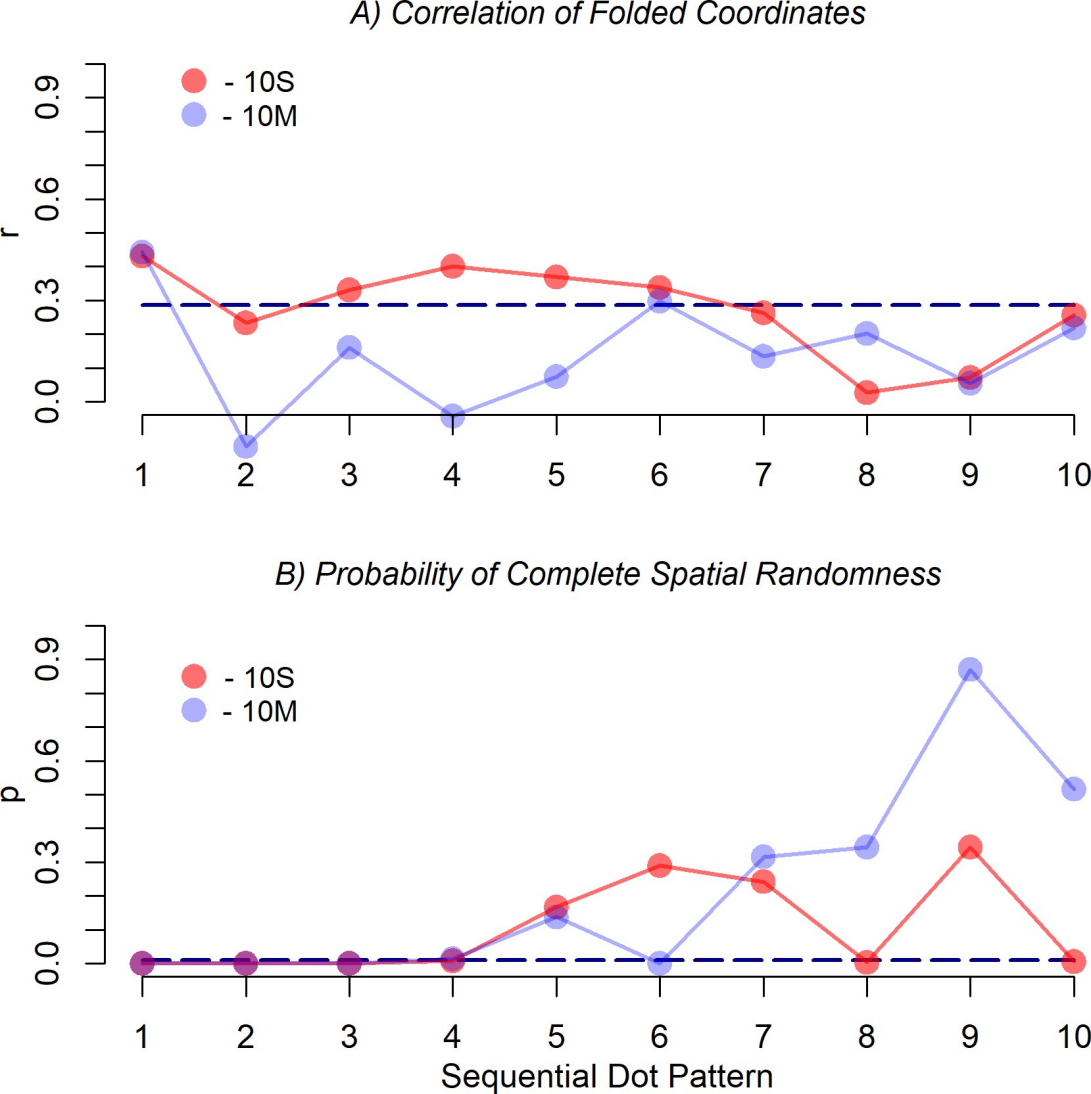
Correlations of the two-times folded coordinates (panel *A*) and *p*-values of the quadrat tests (panel *B*) for the successive ten superimposed dot patterns in the two conditions in Experiment 3. The dashed line in the upper graph indicates when a correlation is significant. In the lower panes it indicates *p* = 0.01.

The correlations of the two-times folded dot coordinates are shown in panel *A* of Fig 13. As can be seen, the correlation is highest for the first dot, which holds for both conditions (10S: *r* = .431; 10M: *r* = .443). The correlations for the next dot are lower and not significant. However, for further dots they rise and fall again.

The *p* values of the CSR test (5×3 grid) for each pattern and condition are shown in panel *B* of Fig 13. Obviously, in both conditions, participants placed the first four dots in locations also preferred by many others. Only then the selected locations began substantially to differ.

### Discussion

This experiment was similar to the first one, except that the canvas was horizontally extended by one third. Consequently, the diagonals of the resulting rectangle were less steep and no longer perpendicular to each other. One question was whether the diagonals are again most attractive for placing dots as in Experiment 1 with a square area, or whether the dots cluster more along a Müller-Lyer figure as in former studies [22, 41]. If we consider Fig 9, then it is obvious that the results are rather similar to those in Experiment 1. In the single-dot condition the dots cluster in the center, along the diagonals, and along the axes. They are more broadly distributed in the mulitple-dots condition. However, in both conditions the empty outer regions on the left and right side are much larger compared to Experiment 1, presumably due to the increased width. This shows that the participants did not use the extra horizontal space for placing dots, except along the diagonals. Thus, Fig 9 looks like a stretched version of Fig 1.

The similarity of the dot patterns to those in Experiment 1 allowed to use the same model for estimating the map of regional attraction. However, different from the quadratic pattern for the multiple-dots condition, the axes were no longer attractive.

The mean path length was again larger in the single-dot condition. With respect to the directions, there were again many paths with an orientation close to the diagonals. However, as confirmed by computer simulations, the percentage of real random lines is also higher for the diagonal directions. This follows from the different ranges of possible lengths for the different directions. Accordingly, paths with a horizontal direction are also more frequent than those with a vertical direction. Compared to these expected percentages, only the down-left and down-right directions are significant more frequent in the single-dot condition. For the multiple-dots condition, only paths with a right and down right direction occurred more frequently.

If we consider the superimposed patterns for the individual sequential dots (Fig 12), then they are also similar to those in the first experiment. The pattern of the first dot is rather different from that of all dots in a condition. Again, most participants placed the first dot along the minor diagonal in the upper left quadrant. This was also independent of the order of conditions. When the single-dot condition came first, 26 of the 38 participants in that condition placed the first dot in upper left quadrant, and 22 did this in multiple-dots condition. The corresponding numbers for the case where the multiple-dots condition came first are 35:43 (10M) and 25:43 (10S). This shows that the general tendency was to start the task with placing a first dot along the minor diagonal in the upper left quadrant. The correlations between the two-times folded dot coordinates indicate, similar as in Experiment 1, a negative recency effect, i.e., that the second dot was placed off the diagonal, which was more pronounced for the multiple-dots condition. Nevertheless, the first four dots were then placed in commonly preferred regions, similar to Experiment 1.

Taken together, the results of this experiment with a rectangular area are rather similar to those for the square area in the first experiment. Concerning the form of the hidden structure, neither the superimposed pattern of the first dot nor that of all dots in a condition looks similar to the Müller-Lyer form.

## General discussion

In the present study, it was investigated how position biases affect the selection of locations within a predefined rectangular area. Many previous studies have examined such biases by asking participants to select an item from a menu, a suspect from a police lineup, or numbers on a lottery ticket [5]. However, selecting such items is not only affected by their position, but sometimes also by their other features [13]. For instance, the probability that a certain number on a lottery ticket is selected also depends on its representativeness [15, 36]. Therefore, to minimize such non-spatial influences, participants in the present study had not to choose items from an array, but to produce dots at random locations in an empty rectangular area. From previous studies it is already known that people have severe difficulties to select locations randomly. Rather, they prefer specific regions. In art theory [16, 17] it has been proposed that these preferences reflect the hidden structure of emergent perceptual forces within an area.

The present study has not only investigated such hidden structures of attraction in detail but went further by applying new methods of data collection, analyses, and modeling. Moreover, processes of sequential location selection have been examined under different conditions. The general task for the participants was to place dots at random locations in a rectangular area by clicking with the computer mouse at corresponding positions on the screen. As all people are familiar with rain, and raindrops are distributed according to a homogeneous spatial Poisson point process [49], the participants were instructed to imagine raindrops falling on a corresponding area. There were two main conditions, each of which required to sequentially select ten locations (Experiments 1 and 3). In a single-dot condition, each dot had to be placed in an empty area, respectively, whereas in a multiple-dots condition the already placed dots remained visible.

The results of Experiment 1, in which the area had a square shape, demonstrate that people do not place dots at random locations. Rather, the patterns of the superimposed dots across all participants show that certain regions are preferred. In both conditions more dots were placed in the upper half of the area than in the lower one. This result is not only similar to that in Lisanby and Lockhead [22], who used a method comparable to that used in the present study, but also to the top-row bias observed in choosing a suspect from a photo array [7]. Furthermore, regions at the center, the diagonals, and the principal axes were preferred, which also replicates previous results [e.g., 22], and is in line with ideas from art theory [16, 17]. Thus, basic results previously obtained with paper and pencil were reproduced in the present online study by using computer technology.

To make the spatial structure of these position biases more visible, intensity maps were computed from the dot patterns. They indicate the average number of dots per unit area expected from a spatial Poisson process in that region. The intensity maps allowed to recover the hidden structure of regional attraction. By assuming that the attraction of the center, the diagonals, and the axes has a certain individual form of a bivariate normal density distribution, respectively, it was possible to construct a theoretical map of regional attraction that predicts very well the expected frequency of placed dots. Specifically, for both conditions these maps explain more than 75% of the variance of the intensities estimated from the dot patterns. This value is encouragingly high, considering that results such as more dots being placed in the upper half of the area are ignored by the model.

In Experiment 1 it also became clear that superimposed dots are more widely distributed in multiple-dots conditions than in single-dot conditions, indicating a less modulated regional attraction. This result is in line with previous results [34, 43] and compatible with the general hypothesis that the variety of simultaneous decisions is larger than that of sequential ones [50, 51]. Possible reasons for the results are that visible dots have a repelling effect [17], or simply that the visibility of previous dots make inhomogeneous distributions more apparent. Interestingly, sequential analyses of location selection revealed that the visibility of dots significantly reduced the mean distance between successive dots, compared to the single-dot condition. This suggests that in the multiple-dots condition the participants tried to construct a whole spatial pattern that looks random, whereas in the single-dot condition they tried to produce a random series of dots. This account is further supported by the fact that the direction of the paths also differed between the conditions. In the single-dot condition participants frequently moved from one dot to the next along some of the diagonal directions. This was not the case in the multiple-dots condition, where the movement was often in the horizontal direction. Importantly, the direct paths between subsequent dots were also longer in the single-dot condition. Together, these results suggest that the visible dots in the multiple-dots condition exerted some local repelling effect, while in the single-dot condition the memorized location of the previous dot motivated the participants to place the next dot further away, which can be considered as some kind of negative recency effect. In any case, these results indicate that the conditions induced specific planning strategies.

Planning differences between the conditions are also obvious if the sequence of superimposed dot patterns is considered. In the multiple-dots condition about two thirds of the participants placed the first dot in the upper left quadrant of the area, and mostly along the minor diagonal. In this respect the results differ from that in previous studies, where the structure, obtained here after superimposing all dots, was already observed for the first and only dot. This indicates that, if participants know they have to place only one single dot, their location selection is more heterogeneous, but nevertheless affected in a similar way by the overall regional attraction as in the present case. This is reminiscent of the differences in position bias between answering an isolated single multiple-choice question and a full length test [9].

The fact that in Experiment 1 most participants started near the minor diagonal in the upper left quadrant is also indicated by the high correlation between the two times folded x and y coordinates of the first dot. Usually, the correlation indicates a high intensity along all diagonals. However, the present results show that a high correlation does not guarantee that dots are present anywhere along the diagonals. It is sufficient if dots frequently occur only along a part of a diagonal. In any case, here the high correlation means that the first dot was frequently placed along the minor diagonal in the left upper quadrant. The next dot was then often placed near the center. Across the ten sequential placements, the correlation of the two-times folded coordinates of the dots rises and falls like a damped wave, which indicates that people systematically switch between a diagonal region and an off-diagonal one. This sequential pattern is much less pronounced in the multiple-dots condition.

The sequential analyses of the intensity maps for the individual dots show a supplementary view of the strategy. They revealed that the participants select commonly preferred regions throughout the first dots but then diverge. Thus, at the beginning participants seem to follow a common order of regions. To what extent this order reflects an order of preference is hard to tell. It is also possible that it exhibits a reading or writing habit. That such a habit might have been involved, is suggested by the fact that there were many horizontal paths with a direction from left to right.

For assessing whether the observed dot patterns are random or not, CSR (complete spatial randomness) tests were computed, which provide the probability that a pattern results from a homogeneous Poisson point prosses [44]. As mentioned, a Poisson process is characterized by homogeneity (there is no preference for any spatial location) and independence (the outcome in one region has no effect on the outcome in other regions). The latter property requires a random number of dots. Because of the number of dots was fixed in Experiment 1, which implies that the knowledge of the number of dots in one region determines the number of dots in the remaining region, independence was not given. Therefore, the corresponding random process is actually a binomial point process. To examine whether the violation of the independence property is crucial for the present objective, particularly for the validity of the CSR test, a binomial point process was simulated for each condition by distributing the corresponding number of dots uniformly over the area. As result, whereas the empirical patterns significantly deviate from a Poisson process, this is not the case for the random pattern, suggesting that the independent assumption is not crucial for the present objective.

Nevertheless, to also approach this problem more directly, a second experiment was conducted with two multiple-dots conditions. In one of these (30M), the participants had to place 30 dots, whereas in the other (XM) they were free to place as many dots as they wanted. As a result, in condition XM the participants produced a large range of dots from 1 to 158. Interestingly, they summed up across participants to a similar number as in condition 30M. Most importantly, though, although condition XM would have allowed to mimic a true Poisson process, this is not what the participants did. Rather, the superimposed dot patterns were rather similar between the two conditions. Compared to Experiment 1, more dots were placed in the outer regions. However, the regions near the borders were still largely ignored. In any case, the CSR test is significant for both conditions, suggesting that the assumption of a random number of dots is not crucial for the present data.

To investigate whether the results obtained so far for a square area also hold for a rectangular area, a third experiment was conducted in which the horizontal dimension of the area was extended by one third. Former studies found that the regions of attraction differ between rectangular and square shapes [22, 41]. Rather than placing dots along the diagonals, as in a square area, they were placed in rectangular areas along the inward pointing half of the Müller-Lyer illusion [48]. However, although the width to height ratio in Experiment 3 was of 1.5, which is rather similar to that of 1.47 in Lisanby and Lockhead [22], the result was comparable to that in Experiment 1. Accordingly, the present study does not confirm that the structure of attraction qualitatively differs between areas with a square and those with a rectangular shape. A possible explanation for the inconclusive results could be that the former studies used paper and pencil, where the hand might have covered part of the area. Moreover, the sheet for placing the dots was flat rather than upright. However, it is unlikely that these methodological differences are crucial. It is more reasonable that the requirement to place several dots in succession, compared to only a single dot as in the other studies, is critical. The task of randomly placing several dots motivated the participants to plan the sequence of locations and/or the pattern ahead. This planning not only led to similar sequential dot patterns for square and rectangular areas but was probably also responsible for the similarity between the areas of attraction.

Taken together, the results of the present study again show that the selection of locations in a given area is systematically biased. If people have to place dots at random locations within a rectangular area, they are largely attracted by the center, the diagonals, and the axes of that area. The regional modulation of attraction is somewhat reduced when previously placed dots remain visible. Nevertheless, both conditions can well be modeled by the same relatively simple model of regional attraction. Concerning the shape of the area, no qualitative differences were observed between a square and a rectangular area. Moreover, not only the superimposed dot patterns were similar but also the sequential patterns. They indicate that the placement of successive dots was planned ahead and surprisingly systematic rather than random. Moreover, the planning substantially differed depending on whether the already placed dots remained visible or not.
